# Volcanogenic Fluvial-Lacustrine Environments in Iceland and Their Utility for Identifying Past Habitability on Mars

**DOI:** 10.3390/life5010568

**Published:** 2015-02-16

**Authors:** Claire Cousins

**Affiliations:** UK Centre for Astrobiology, School of Physics and Astronomy, University of Edinburgh, Mayfield Road, Edinburgh EH9 3JZ, UK; E-Mail: c.cousins@ed.ac.uk; Tel.: +44-131-651-7771

**Keywords:** Mars, lacustrine, fluvial, volcanism, habitability, Iceland, astrobiology

## Abstract

The search for once-habitable locations on Mars is increasingly focused on environments dominated by fluvial and lacustrine processes, such as those investigated by the Mars Science Laboratory *Curiosity* rover. The availability of liquid water coupled with the potential longevity of such systems renders these localities prime targets for the future exploration of Martian biosignatures. Fluvial-lacustrine environments associated with basaltic volcanism are highly relevant to Mars, but their terrestrial counterparts have been largely overlooked as a field analogue. Such environments are common in Iceland, where basaltic volcanism interacts with glacial ice and surface snow to produce large volumes of meltwater within an otherwise cold and dry environment. This meltwater can be stored to create subglacial, englacial, and proglacial lakes, or be released as catastrophic floods and proglacial fluvial systems. Sedimentary deposits produced by the resulting fluvial-lacustrine activity are extensive, with lithologies dominated by basaltic minerals, low-temperature alteration assemblages (e.g., smectite clays, calcite), and amorphous, poorly crystalline phases (basaltic glass, palagonite, nanophase iron oxides). This paper reviews examples of these environments, including their sedimentary deposits and microbiology, within the context of utilising these localities for future Mars analogue studies and instrument testing.

## 1. Introduction

As our search for Martian habitability progresses, so does our understanding of past environments and surface processes on Mars. From the “follow the water” approach employed by NASA during the Mars Exploration Rover and Phoenix missions, Mars exploration has since become focused on the identification of “habitability” and the detection of potential biosignatures [1,2]. One of the greatest shifts in our understanding of Mars surface geology has been the identification of widespread alteration minerals through orbital data from the Compact Reconnaissance Imaging Spectrometer for Mars (CRISM) and Observatoire pour la Minéralogie, l’Eau, les Glaces et l’Activité (OMEGA) instruments [3,4,5], complimenting pre-existing morphological evidence for hydrological surface activity throughout the Noachian and into the Hesperian [6]. The global distribution of these deposits implies that aqueous processes and environments were active across much of the planet, resulting from a range of possible scenarios including climatic hydrological cycles, punctuated episodes of hydrological activity initiated by localised cryospheric melting, release of pressurized subsurface water aquifers, and isolated volcanic and impact events [6,7,8]. The nature of habitability on Mars is therefore underpinned by these processes at both spatial and temporal scales, and includes the availability of bioessential elements, metabolic redox couples, and stability and longevity of liquid water environments [9,10]. The expansive and diverse sedimentary record on Mars ranges from open and closed basin systems to rhythmite deposits putatively linked to climatic cycles, all of which have yet to be definitively explained but provide a record of potentially habitable palaeoenvironments [11]. Deltaic fan deposits for example, particularly those thought to have been deposited within a stable lacustrine setting, such as the Jezero crater open-basin lake system [12], suggest long-lived liquid water environments. Conversely, recent experimental work suggests many delta fans on Mars formed during short, intense episodes of high hydrological discharge [13]. Extensive networks of branching fluvial channels (predominantly Noachian terrains) and large outflow flood channels (predominantly late Hesperian terrains) also characterize the Martian surface [14]. Continued valley formation into the early Amazonian has been identified, with the youngest valleys thought to be related to hydrothermalism due to their confinement to volcanic constructs [15]. Similarly, alternating episodes of aqueous flooding and volcanism identified at Mangala Valles potentially indicate a causational relationship between the two, with evidence for repeated and interacting subsurface igneous and hydrological events occurring into the Late Amazonian [16]. Recently, [17] demonstrated the potential for short periods of global climatic warming instigated by episodic flood volcanism throughout the Noachian and Hesperian, which would result in temporary melting of surface ice and snow and the generation of fluvial and lacustrine systems.

Understanding the cause and characteristics of these aqueous environments for astrobiology is challenging, but regardless of their interpretation, the deposits of such fluvial and lacustrine settings are currently a major target in continued robotic exploration of Mars, including the continued exploration of Gale Crater by Mars Science Laboratory and ongoing site-selection for the ESA ExoMars 2018 rover mission. Therefore, identifying which microbial metabolisms are supported by episodic, basalt-hosted fluvial-lacustrine systems can inform what biosignatures to search for, and how well rover instrumentation fares when deployed on such deposits. Likewise, understanding the preservation and detection of organic carbon and other “ingredients” of habitability within phyllosilicate- and sulfate-bearing sedimentary deposits that are mineralogically and geochemically analogous to those on Mars is imperative to continued successful exploration. Clay-bearing lithologies have been identified as particularly important targets for the detection of preserved organic matter [18]. On Earth, plate tectonics combined with continual surface and suboceanic weathering results in a complex and evolved clay cycle, whereby neoformed and inherited clays are deposited within a variety of active- and passive-margin sedimentary basins and subsequently buried, transformed through diagenetic and metamorphic processes, and recycled [19]. The lack of plate tectonics on Mars will most likely result in different pathways for clay formation and deposition [20], with the dominance of Fe/Mg clay deposits over Al-rich phyllosilicates consistent with the alteration of basaltic crust at low water/rock ratios [20]. This is consistent with basaltic crust being particularly susceptible to alteration and clay mineral formation due to the low-temperature instability of basaltic mineral phases, and the amorphous nature of any basaltic glass produced via eruptive products at the surface (*i.e.*, lava flows, ash). Therefore, terrestrial environments typified by neoformed, authigenic clays (e.g., volcanogenic and hydrothermal clays, and clays directly precipitated from solution) and detrital inherited clays that have undergone little thermal transformation [19] are useful analogues to clays on Mars. Furthermore, where such clays on Earth form and are deposited in association with environments typified by relatively low biomass, there is scope to establish how biogenic organic matter may be preserved.

Sedimentary systems that exist within polar and high altitude environments are of use to Mars analogue research due to the minimal influence from vegetation, organic-rich surface soils, and anthropogenic activity. Alluvial fans in the Atacama Desert [21], debris flows in Alaska [22], and impact crater lake sedimentation [23] and saline springs [24] within the Canadian High Arctic have similarly been used or proposed as Mars analogues, and have provided important constraints as to the nature of comparable processes on Mars. However, a fundamental difference between the mineralogical products of alteration and weathering and the resulting sedimentary deposits is the relatively evolved, Si-Al rich nature of the Earth’s crust compared to the Fe-Mg rich crust of Mars [25] Initial results from the first four rocks examined by the NASA Mars Science Laboratory (MSL) rover Curiosity revealed three of the four rocks to be volcaniclastic sediments of igneous origin, with a broadly basaltic composition [26], while sedimentary rocks at Yellowknife Bay were also derived from sources compositionally consistent with basaltic crust [27], including detrital mafic minerals (e.g., Fe-forsterite, plagioclase, pigeonite, and augite) within the sediments [28]. This is unsurprising given the largely basaltic composition of the Martian crust [29], and likely to be a factor for other fluvial-lacustrine systems beyond those explored in Gale Crater. Indeed, some areas of extensive alteration on Mars have been interpreted as long-term aqueous interaction with basaltic crust [6] such as through low temperature, neutral pH hydrothermalism [30], high temperature acid fumarole alteration [31], or impact-generated hydrothermalism [32]. Therefore, when using terrestrial-based analogues for fluvial and lacustrine environments on Mars, it is imperative that future instrument testing and biosignature research includes similar basalt-derived sedimentary lithologies. For example, the utility of low-temperature alteration assemblages within the basaltic Deccan Traps, India, as an analogue for Al-phyllosilicate and Fe/Mg smectite stratigraphy on Mars has been previously demonstrated [33].

## 2. Iceland

**Figure 1:**
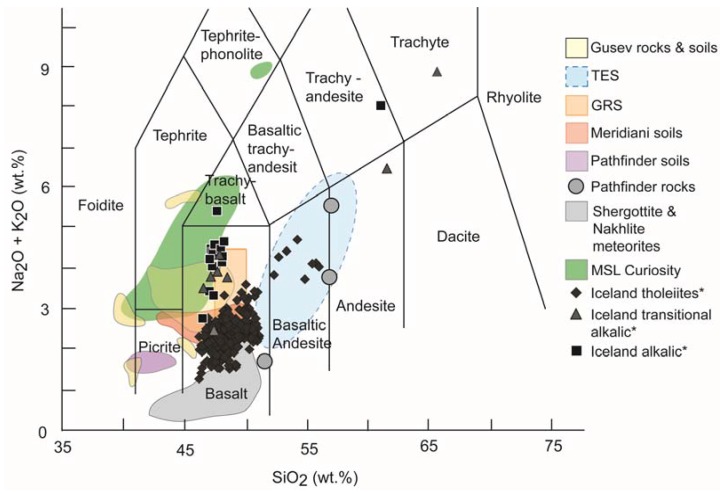
Total Alkali Silica plot adapted from [29] TES = Thermal Emission Spectrometer, GRS = Gamma Ray Spectrometer and including MSL Curiosity APXS data (Tables S1–S3, from [26]; Table 7 from [27]) and supplementary data (* marked) from [35]. All additional data added are 100%-normalised volatile free values.

The volcanic island of Iceland is unique in that its volcanotectonic setting lies at a juxtaposed mantle plume and rift system [34] within a near-arctic location. This volcanotectonic setting results in the crust having a broadly basaltic composition (Figure 1), with tholeiitic eruptive products reflecting these two main magmatic sources within the active rift zone [35,36]. Overall, the majority of Holocene basalts comprise three dominant magma series from tholeiitic, transitional alkalic, to alkalic [35,36], with Pleistocene basalts (predominantly subglacially-erupted pillow lavas and volcaniclastics, and interglacial lavas) exhibiting much the same trend [36,37]. This provides excellent examples of volcanogenic fluvial–lacustrine terrains, deposits, and environments where the primary crust and sediment parent material is basaltic in composition. The combination of Fe-Mg rich and Al-Si poor crust with aqueous environments that are locally influenced by elevated sulfur input due to their proximity to (or interaction with) volcanic activity make these sites valuable field models for detrital and authigenic mineral assemblages and the biosignatures deposited and potentially preserved.

**Figure 2:**
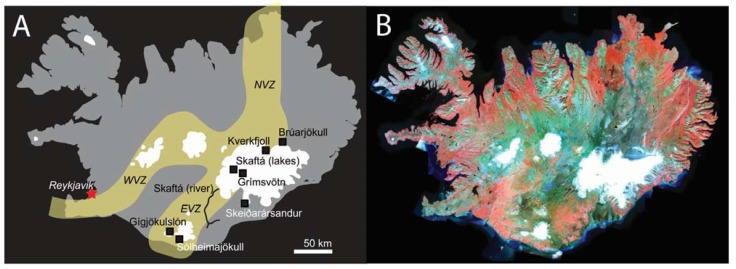
(**A**) Map of Iceland showing the location of the sites covered in this paper. The active north, east, and western neovolcanic zones are shown (yellow), as well as ice cover (white); (**B**) Corresponding National Land Survey of Iceland (NLSI) infrared satellite image of Iceland (IS 50V database/SPOT data), showing the lack of vegetation cover (red) within the neovolcanic zones.

Due to the sub-arctic location of Iceland many parts of the country lie within glacial-periglacial-semi-arid climates, with annual precipitation as low as 400 mm in the interior [38]. As such, an estimated 60% of Icelandic glaciers overlie active volcanic systems [38]. This interaction between active volcanism along the neovolcanic zone and surface ice over the past 0.8 Ma [34] has resulted in widespread volcaniclastic sedimentation, including ubiquitous hyaloclastite/hyalotuff sequences, volcanogenic fluvial and lacustrine environments, and crustal hydrothermal alteration (both low and high temperature). Furthermore, many deposits and ongoing active environments are relatively undisturbed or reworked by vegetation, fauna, or human influence, particularly along the active rift zone (Figure 2B). Moreover, these regions are also specific analogues for putative glaciovolcanic landforms on Mars (e.g., [39]), which may represent one of the few habitable environments that could have pervaded into the Amazonian [39,40]. For more detail on the habitability of such glaciovolcanic habitats, the reader is referred to [41,42]. Iceland is a well-known locality for Mars analogue research, including aqueous alteration of basaltic crust [43] and gully formation [44]. Volcaniclastic sedimentary terrains and the fluvial-lacustrine processes that modify them have been largely overlooked, although comparisons have been made previously between subglacial outflow events (“jökulhlaups”) and flood channels on Mars [45], and a study by [46] demonstrated the utility of low temperature alteration phases within Icelandic basaltic outwash sediments as a good analogue for detecting similar assemblages on Mars. This paper provides a synthesis of example sedimentary fluvial-lacustrine terrains and contemporary active lacustrine environments in Iceland, with the view to present these as new and currently underused analogues for similar habitats and deposits on Mars, particularly with relevance to habitability and biosignature detection. As such, the aim of this paper is not to provide a direct geomorphological or formational analogue to specific terrains on Mars, but to expand the current variety of terrestrial localities which are of benefit to ongoing and future robotic exploration of Mars.

This paper focuses on five sites located within and around the Vatnajökull ice cap (Brúarjökull, Grímsvötn, Kverkfjöll, Skaftá, and Skeiðarársandur), and two sites in the south at Gígjökulslón and Sólheimajökull, as shown in Figure 2. These sites include examples of subglacial, englacial, and proglacial lacustrine environments and sedimentary deposits, and proglacial fluvial sedimentary deposits including jökulhlaup deposits, sedimentary fans, and sandur plains. These sites are described regarding their sedimentary deposits and processes, microbiology, and potential for future Mars analogue research, summarized in Table 1.

**Table 1:** Volcanogenic fluvial-lacustrine environments and sedimentary deposits discussed in this paper and their utility for Mars analogue research.

Field Sites	Mars Analogue Investigations
Instrument Testing	
Brúarjökull proglacial region Kverkjökull sandur Skeiðarársandur and Sólheimajökull	Large-scale field testing of context and close-up imaging instrumentation to positively identify large-scale stratigraphy and depositional fabrics, lithofacies, and small scale sedimentary structures, diagenetic features, and associated chemical composition (e.g., corroboration with spectroscopic data along a stratigraphic sequence). Testing sample acquisition and manipulation for caching or processing for analytical instruments, and spectroscopic, mineralogical, and geochemical instrument testing on sediments sourced from basaltic terrains.
Alteration Mineralogy	
Brúarjökull proglacial region Skeiðarársandur and Sólheimajökull Gígjökulslón	Low temperature hydrothermal and pedogenic alteration of primary basalt, particularly investigating authigenic *vs.* detrital alteration minerals within combined fluvial-lacustrine systems, and precipitation of neformed clays from recent (<5 years) basaltic sediments and glass-rich volcanic ash.
Kverkfjallalón	Low-high temperature hydrothermal alteration of basaltic sediments and resulting hydrated mineral assemblages (e.g., [41], and how such alteration phases are fluvially-transported and deposited.
Biosignatures	
Brúarjökull proglacial region Skeiðarársandur and Sólheimajökull Gígjökulslón	Preservation and detection limits of organic biosignatures within clay-poor lacustrine environments, particularly on the influence of age and lithification on the preservation of biosignatures. Detection limits of trace organic deposition within a short-lived proglacial lacustrine environment.
Kverkfjallalón	Preservation and detection limits of isotopic biosignatures within smectite clay-bearing lacustrine sediments, particularly as a depositional system for seasonal sulfate-dominated hydrothermal streams [41].
Hveragil stream	Successive mineralisation of biogenic organic matter within hydrothermal fluvial carbonate deposits.
Skaftá western and eastern lakes	Identification of biogenic organic productivity and burial within low-temperature, sulfidic lacustrine systems, and the geochemical biosignatures generated through chemotlithoautotrophic metabolisms (e.g., carbon and sulfur stable isotope fractionation patterns), and tracing these biosignatures from subglacial microbial communities (source) to deposited sediments (sink).
Microbiology	
Skaftá western and eastern lakes	Anaerobic metabolic pathways based on sulfide and CO_2_, adaption to extremes (oligotrophy, cold temperatures, environment instability), and biogeochemical cycling of CHNOPS in lacustrine environments.
Hveragil stream	Microbial communities within a seasonal CO_2_-rich fluvial environment fed by subsurface hydrothermal fluids and glacial meltwater from Kverkfjöll volcano.
Kverkfjallalón Galtarlón Grímsvötn	Adaption to extremes including oligotrophy, seasonal ice-cover, and biogeochemical cycling of CHNOPS within young and transient lacustrine environments. Elucidation of viable metabolic redox couples and their response to limiting CHNOPS.

## 3. Lacustrine Environments

Lakes in glacial settings can form as a direct result of volcanic activity, where ice is converted to water by an increase in geothermal heat, forming englacial (glacier-bound), supraglacial (occurring at the glacier surface), and subglacial (occurring beneath the glacier) lakes. Because glacier margins themselves can also be sites of lacustrine activity (e.g., proglacial lakes), there is a complex interrelationship between volcanic episodes, lake evolution, and the resulting sedimentary products [47]. The volcanogenic glaciolacustrine environments detailed here result from the direct interaction between either a central volcano or hydrothermal vents and overlying ice [48]. They range from active subglacial lakes at Grímsvötn and Skaftá, and englacial hydrothermal lakes at Kverkfjöll, to glaciolacustrine sedimentary deposits north of Brúarjökull.

### 3.1. Subglacial Lakes

**Figure 3:**
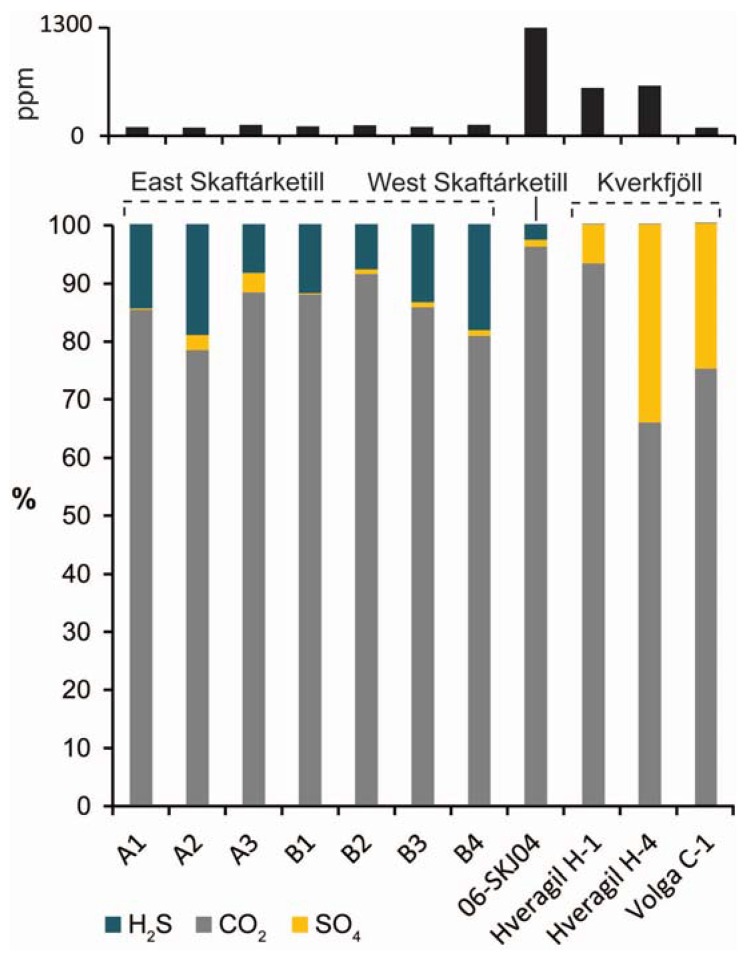
Proportion of dissolved CO_2_ (77–1300 ppm), H_2_S (0.03–36.9 ppm), and SO_4_^2−^(1.03–42.1 ppm) within East (A1–B4, data from [55]) and West (06-SKJ04, data from [53]) Skaftá subglacial lakes, and within the river Volga (Volga-C-1) and Hveragil (H-1 and H-4) outflows at Kverkfjöll (data from [55]. Upper plot shows total concentration for each respective site.

Vatnajökull ice cap overlies several active or dormant central volcanic systems within the neovolcanic zone [49]. Of these volcanic systems, Grímsvötn has been regularly active, producing recent eruptions in 1996 (Gjálp), 1998, 2004 and 2011. The eruption observed in 1996 at Gjálp was entirely subglacial, resulting in meltwater ponding beneath the ice cap [50]. This process is common at volcano-ice interaction sites, with ongoing heat flux often maintaining a subglacial meltwater lake in between eruptions [51]. Several investigations have been conducted into the biogeochemical environment and indigenous microbiology of the subglacial lakes at Grímsvötn and at the nearby Skaftá lakes beneath Vatnajökull. In 2004 [52] sampled the subglacial lake confined within the Grímsvötn crater ~300 m beneath the ice surface and identified a viable microbial community residing within the lake. Results showed the −0.2 °C, oxic, mildly acidic (pH 4.87–5.13) lake supported a community of psychrotolerant bacteria, distinct from bacterial communities within the surrounding ice and snow, that was well adapted to a glacial environment. In contrast, the nearby geothermal subglacial lake at Western Skaftá forms a warmer (3.5–6 °C) anerobic environment within its bottom waters, with a measureable input of hydrothermal fluids into the lake [53]. As such, the microbial communities indigenous to the subglacial anoxic bottom waters here are dominated by obligate or facultative anaerobes including members of *Acetobacterium*, *Thermus*, *Paludibacter*, *Sulfuricurvum*, *Pseudomonas*, and *Sulfurospirillum* species, forming a microbial ecosystem potentially driven by sulfide oxidation, sulfate reduction, and hydrogen oxidation [54] utilizing the available CO_2_ and sulfide within the lake environment (Figure 3). A similar geochemical environment (Figure 3) and microbial community was identified at the Eastern Skaftá subglacial lake, with anoxic water characterized by mean dissolved H_2_S of 16 ppm and CO_2_ of 105 ppm [55] and a microbial population dominated by *Acetobacterium*, *Geobacter*, *Sulfurospirillum*, *Sulfuricurvum* and *Desulfosporosinus* species.

Further investigation into a jökulhlaup outflow along the Skaftá river and a subaerial englacial lake (Kverkfjallalón—discussed in the next section) has shown that certain taxonomic groups here share a high level of genetic similarity with those in the subglacial Western Skaftá and Eastern Skaftá lakes, suggesting these systems are fed by a deeper crustal biosphere interconnected via groundwater circulating within the permeable basaltic crust beneath Vatnajökull, with differences in community composition controlled by local environmental conditions [55]. The chemolithoautotrophic sulfur oxidizing bacterium *Sulfuricurvum* in particular was found across all environments [55]. Such interconnected subsurface microbial habitats driven by episodic volcanogenic hydrothermal input, are analogous to habitable environments that feasibly may have existed on Mars [40], particularly at sites where direct volcano–ice interaction has been identified, such as Arsia Mons [39,40].

### 3.2. Englacial Lakes

**Figure 4:**
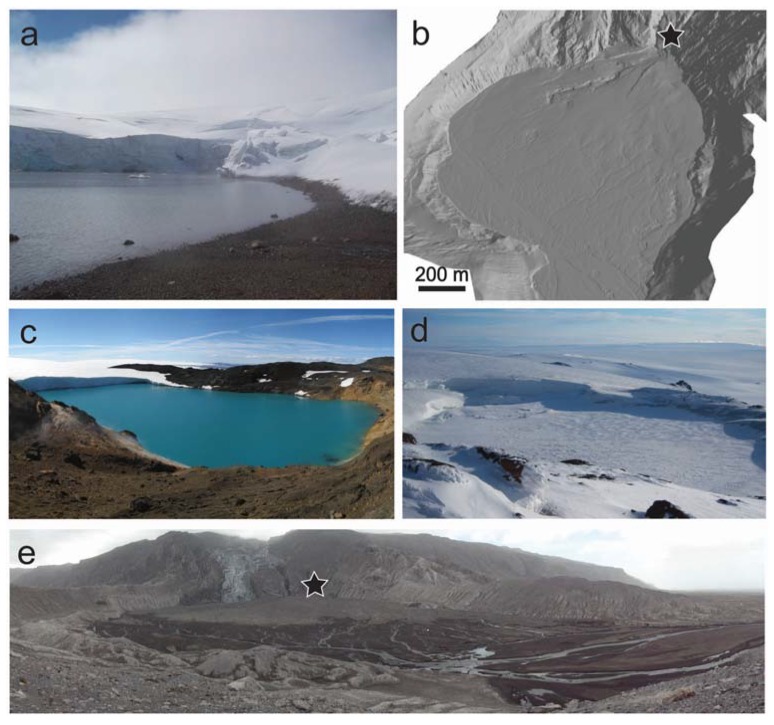
Examples of lacustrine environments and deposits. (**a**) Subaerial lake Kverkfjallalón (2011) surrounded by sulfate and smectite-rich sediment [41]; (**b**) Hillshaded terrestrial laser scanner image of the sediment fan at Gígjökulslón, 2010 following the eruption of Eyjafjallajökull (image credit: Stuart Dunning) [60]; (**c**) Galtarlón, ice free, July 2007 (image credit: Katherine Joy), lake approximately 300 m across at its widest point; (**d**) Galtarlón, ice covered, June 2011 (image credit: Barry Herschy); (**e**) Oblique view of the Gígjökulslón sedimentary fan looking towards the fan source, marked by black star (image credit: Stuart Dunning, [60]).

Ice-bound englacial lakes sustained by hydrothermal activity provide a subaerial counterpart to the subglacial lakes detailed above, which are more readily accessible for sampling and research. Hydrothermal input into these lakes includes active hot springs and fumaroles within and around the shore of the lake [56]. At the Kverkfjöll volcano on the northern margin of Vatnajökull, the ~300 m diameter englacial hydrothermal lake Kverkfjallalón, locally also named Gengissig, is sustained by geothermal heating in between periodic drainage events, the most recent of which occurred in August 2013, producing a small jökulhlaup following a phreatic event [57]. Geothermal heating of this lake keeps it seasonally ice-free (Figure 4a), with summertime (June 2011) temperatures between 10–20 °C at the lake shore [41]. The microbiology from one sample taken at 4 m depth within this lake was found to be dominated almost entirely by the aerobic betaproteobacterium *Xenophilus*, a microorganism common to glacial environments [55]. However, for elucidating comparable metabolisms that could have operated within Martian lacustrine environments, where only anaerobic metabolisms are likely to have been feasible, englacial subaerial lakes such as Kverkfjallalón benefit from hydrothermal input, which results in localized anoxia or reduced dissolved oxygen (DO) at points of direct hydrothermal interaction or mixing, contrasting with the oxic areas of the lake. For example, DO at the lake edge measured in 2011 ranged from 0.8–3.9 mg/L [41]. Coupled with the availability of dissolved sulfate [41] (Figure 3), this provides a natural laboratory within which to investigate anaerobic chemolithotrophy within a lacustrine setting as a model for past Martian ecosystems. Indeed, glaciovolcanic landforms at Arsia Mons suggest that such an englacial lake was sustained through volcanic heating, potentially for hundreds to thousands of years [39]. Adjacent to Kverkfjallalón is a younger ice-dammed lake (locally termed “Galtarlón”). This clear blue lake (Figure 4c) has not yet been investigated with regards to its resident biota, but the clarity of the lake water suggests this environment will be highly nutrient limited, similar to supraglacial lakes observed on Greenlandic ice sheets [58]. These lakes represent low temperature hydrothermal lacustrine environments, with geochemical inputs driven by passive leaching of bioessential dissolved ions (e.g., Si, Ca, Na, Fe, Mg) from the underlying basaltic bedrock, mixing with hydrothermal fluids draining into the lake [41,53], and by active volcanic degassing of CO_2_, H_2_S, and H_2_ [56]. One major factor that may have differed between englacial lakes on Earth and lacustrine environments on Mars is the potential presence of sustained ice cover on Martian lakes, and the effect of this on sunlight and delivery of exogenous nutrients. However, given that phototrophy has not been identified as the primary means of production within the indigenous microbial communities present within Kverkfjallalón [55], and that both Kverkfjallalón and nearby Galtarlón typically lose their ice-cover predominantly during summer months (and some years not at all, Figure 4c,d), which has the benefit of improving accessibility for sampling, this difference does not detract from the value of these sites as a suitable microbial analogue.

Sediments sampled from Kverkfjallalón and nearby lake shore areas in June 2011 were found to comprise of detrital plagioclase and pyroxene sourced from the underlying basalt, and alteration phases including smectite clays, gypsum, jarosite, pyrite, heulandite, and quartz [41]. This combination of unaltered basaltic minerals and low temperature alteration phases is analogous to sediments (although not the depositional environment) explored by the MSL Curiosity rover at Yellowknife Bay, which included detrital basaltic minerals, smectites, calcium sulfates, iron oxide or hydroxides, iron sulfides, and amorphous material [28]. Englacial lake sediments can be emplaced in the sedimentary record via repeated jokulhlaup events, such as the jokulhlaup sandur deposits identified in the proglacial region of Kverkfjöll [59] and described in Section 4 below. Over long time scales, englacial hydrothermal lake sediments deposited either via jökulhlaups or through deposition by a receding glacier would be identifiable by the presence of mineral assemblages that include pyrite and other sulfur-bearing alteration minerals, including jarosite and gypsum [41]. Such sediments therefore provide a test environment for tracing the local environmental history and available metabolic redox couples.

### 3.3. Proglacial Lacustrine Environments

Unlike subglacial and englacial lakes, proglacial lakes can be the focus of significant sediment deposition, but may lack the hydrothermal input required to sustain a variety of chemolithotrophic metabolisms. A recent example of lacustrine fan deposition occurred at the 20–30 m deep proglacial lake Gígjökulslón following the 2010 eruption of Eyjafjallajökull [61]. Here, a volcanogenic jökulhlaup sequence was dominated by two large outburst floods totalling 57 × 10^6^ m^3^ of water [60]. Meltwater stored within the glacier drained sub- and supra-glacially into Gígjökulslón, producing two prograding ice-contact sediment fans (Figure 4b,e). Further outbursts of meltwater occurred, completely infilling Gígjökulslón, followed by >140 smaller outburst floods, attributed to continued subglacial melt at the eruption site and beneath Gígjökull glacier [60]. Together, these events resulted in a depositional fan of varying steepness emanating from a bedrock gorge (Figure 4e). The jökulhlaups were sediment rich, draining much of the subglacially-erupted volcanic material [61] with the majority of deposition estimated to occur over a short time frame of ~48 h [60]. Such deposits provide an example of primary, organically-poor basaltic sediment fans that have undergone little aqueous alteration or transport.

The emplacement of volcanic edifices and subsequent localised increase in elevation can also lead to the natural damming of glacial meltwater and subsequent formation of a proglacial lake during glacial retreat. An example of this can be seen in the Pleistocene sedimentary record within the proglacial region of Brúarjökull. Here, a palaeolake damned by the subglacially erupted pillow basalt and volcaniclastic edifice of Kárahnúkar became progressively in-filled with lacustrine and deltaic sediments during deglaciation in the Quaternary [62]. These proglacial lacustrine sediments at originated from subaqueous fan and low-angle delta environments, and are now partly exposed along the Jökulsá á Brú from the current margin of Brúarjökull. Following deposition of these lacustrine sequences, fluvial channels have since cut into these sediments, thought to result from the lake dam being breached [62]. Lacustrine and glaciofluvial sedimentary sequences formed during glacial retreat also exist along the nearby Fljótsdalur and Jökuldalur valleys in East Iceland [63], which channelled ice flow over successive glaciations [64]. This process of sedimentation forms a temporal contrast to the rapid sediment formation and deposition observed during jökulhlaup release into a lake, as observed at Gígjökulslón. These sites therefore contain a sedimentary record depicting a range of depositional lacustrine environments. There is an absence of a clay component here, even within the fine-grained sediment units [63]. While this limits this site for analogue work focusing on those lacustrine environments on Mars that exhibit phyllosilicate spectral signatures, it is more applicable to the majority of paleolake systems on Mars that do not show this evidence for alteration minerals [65].

## 4. Fluvial Environments

Volcanogenic fluvial processes are common in Iceland, where volcano-ice and geothermal-ice interaction can result in the sudden drainage of large volumes of stored glacial meltwater (jökulhlaups) and continual low-discharge meltwater release in between jökulhlaup events [51]. Moreover, geothermal and meltwater dissolution of glass-rich basaltic tephra releases elements into these meltwaters, enabling the delivery of biologically-limiting elements (e.g., phosphorous and iron) into the surrounding proglacial environment [66]. For example, the 1996 jökulhlaup originating from the subglacial eruption at Gjálp had an estimated dissolved volatile and element load of 1 million tonnes [67,68], with suspended sediments comprising fresh glass, palagonite (amorphous primary product of basaltic glass alteration), alteration minerals (such as zeolite and calcite), plagioclase, augite, and olivine, and rock fragments [67]. This meltwater generated by the Gjálp eruption had a pH ranging between 3 and 8 at the eruption site, with the resulting flood water pH ranging from 6.88 to 7.95 [68]. Jökulhlaups from Mýrdalsjökull and Vatnajökull in 2011 were also found to have alkaline-neutral pH waters [66], with increased levels of dissolved organic carbon, formate, and acetate observed within the Mýrdalsjökull jökulhlaup, which suggests microbiological activity was present within the subglacial meltwater stored beneath Mýrdalsjökull prior to its catastrophic release [66]. This is consistent with the observation of viable microbial communities within subglacial lakes at Grímsvötn and Skaftá cauldrons beneath Vatnajökull [52,54,55], and highlights the potential for subglacial meltwater release as a mechanism of transport and deposition of biogenic organic matter within volcanogenic fluvial and lacustrine fan sediments. A contemporary example of this process is provided by the multiple jökulhlaups (at least 40 since 1955 [48]) that have occurred along the Skaftá river, draining the Western and Eastern Skaftá subglacial lakes [48], some of which are known to be inhabited by chemolithotrophic bacterial communities [54,55]. A study by [69] showed high levels (nearly 9 g·L^−1^) of suspended fine-grained sediment (up to ~60% sediment 0.002–0.02 mm in particle size) was transported during a jökulhlaup here in 1997, together with increased concentrations of chloride and fluoride, consistent with geothermal input into the meltwaters [69]. Such repeated deposition of subglacially-sourced sediment provides a contemporary analogue to the transport and burial of subglacially-sourced organics derived from the geothermally-driven microbial communities present with the subglacial caldera lakes. While the vast majority of fluvial sediments on Mars are not jökulhlaup deposits, the utility of these Icelandic sites for Mars analogue research lies in their connectivity to lacustrine environments typified by chemolithotrophic and/or oligotrophic microbial communities, mafic mineralogy and related alteration phases, and subsequent deposition within cold and dry basaltic terrains.

**Figure 5:**
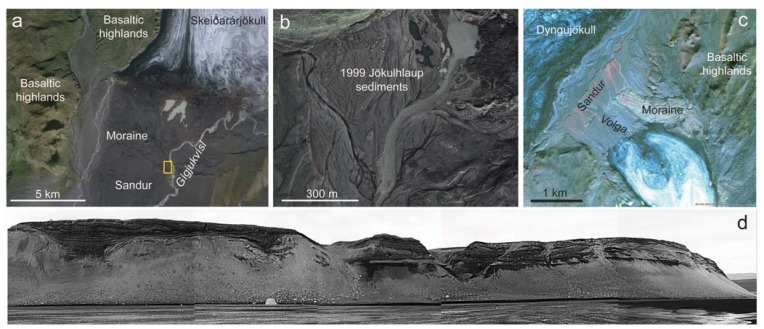
Examples of proglacial fluvial environments and sedimentary deposits. (**a**) Skeiðarársandur, yellow box highlights the location of the fluvial-lacustrine sedimentary succession described in [70], and shown in (d); Image credit: SPOT5/Google Earth; (**b**) jokulhlaup sediments and channels at Sólheimajökull (adapted from [73]). Image credit: SPOT5/Google Earth; (**c**) National Land Survey of Iceland (NLSI) infrared satellite image (IS 50V database/SPOT data) of the Kverkjökull sandur described in [59]; (**d)** Cross section (approx. 240 m long) of fluvial-lacustrine sediments exposed along the Gígjukvísl river [70] at the location marked on (a), image credit Philip Marren.

Further to the south of Vatnajökull, the Skeiðarársandur proglacial sediments form the largest active outwash plain in the world [38], and as such record an extensive history of fluvial-lacustrine sedimentary deposition throughout the Holocene. An assemblage of fluvial and lacustrine sediments [70] is revealed along the Gígjukvísl River (Figure 5a), cut by the jökulhlaup in 1996. The section exposed here is ~240 m long with a maximum height of over 20 m (Figure 5d), and contains seven lithofacies, of which three are identified as fluvial, and four as lacustrine, forming a succession of depositional environments including a shallow ice-marginal braided river, proglacial braided river gravel bed, glaciolacustrine deposits, a high energy subaqueous flow, and finally a jökulhlaup event [70]. This outcrop therefore provides a variety of continuous sedimentary lithofacies for investigating biosignature deposition across a range of environments, and testing of future robotic instrumentation (Table 1). Sedimentary structures within the different fluvial lithofacies range from horizontally stratified or channelized sand layers within the ice marginal shallow braided stream, to well-sorted and cross-bedded gravels in the braided river gravel bed. Glaciolacustrine lithofacies encompass interbedded silts and fine sand and rippled silt layers. Finally, the jökulhlaup deposit encompasses the coarsest sediments with cross-cutting scours cutting into poorly sorted gravels, with well-developed imbrication [70]. Sediments exposed along the Gígjukvísl river have already been used for Mars analogue work investigating the utility of hand-lens imager type datasets (e.g., similar to data produced by the Mars Hand Lens Imager (MAHLI) on NASA’s MSL Curiosity rover) in characterizing sedimentary grain size and morphology, from which fluvial palaeoflows and processes can be interpreted [71]. Fluvial conglomerates comprising of cemented rounded pebbles have since been identified on Mars by the MSL Curiosity rover, providing the first *in situ* evidence of fluvial sedimentary deposition, most likely as part of a distal alluvial fan deposit [72].

Successions of fluvial sedimentation can also be found at Sólheimajökull (a small outlet glacier of the larger Mýrdalsjökull ice cap) and Kverkjökull (a small outlet glacier near Kverkfjöll, on the northern margin of Vatnajökull). At Sólheimajökull (Figure 5b), a jökulhlaup occurred in 1999 following volcanic activity at Katla subglacial volcano, and included meltwater transport both supraglacially, and along the glacier bed, and temporary meltwater storage within ice-marginal basins [73]. Jökulhlaup sedimentation within the glacier produced esker ridges and a large fan at the glacier snout [73] and deposits of fine (clay–silt or finer) sediment on the glacier surface. Glacial retreat since the event has exposed an esker bearing two depositional units: well-sorted cross-stratified gravels and a poorly sorted boulder unit [73]. Glaciofluvial eskers have been proposed to explain many of the sinuous ridges on Mars [74], potentially in association with glaciaolacustrine deposits [74]. At Kverkjökull (Figure 5c), there are a number of active fluvial environments and associated sedimentary and mineralogical deposits. Within the proglacial region of Kverkjökull, 3 km of sedimentary sections within a fluvially-incised sedimentary fan records at least six volcanogenic jökulhlaup events [59]. This sandur comprises several distinctive lithofacies, including (in order of decreasing grainsize) poorly sorted clast- and matrix-supported boulder-gravel facies, a clast-supported gravel unit that dominates the sedimentary section, a pebble-granule-gravel facies with well-defined bedding planes, and finally a sand facies comprising thin (<1 cm) sand layers which form boundary layers between gravel beds [59]. As with the sedimentary sections at Gígjukvísl, these deposits provide an ideal site for testing robotic instrumentation and biosignature preservation on basaltic sediments within a largely vegetation-free terrain (Figure 5c). The Kverkjökull proglacial environment also includes the river Volga, a meltwater stream of subglacial origin with variable hydrothermal input from the Kverkfjoll high temperature geothermal areas [56]. This CO_2_-sulfate fluvial environment contrasts to the CO_2_-sulfide dominated subglacial lacustrine environments under Vatnajökull (Figure 3). Similarly, to the east of Kverkfjöll lies the geothermal river Hveragil, a CO_2_-rich carbonate-depositing neutral-alkaline stream [41,56].

## 5. Conclusions

The environments and depositional settings detailed within this paper can both sustain and potentially preserve evidence of Mars-analogue psychrophilic or chemolithotrophic microbial communities. These basaltic sedimentary lithologies produced through fluvial and lacustrine action, much of which is volcanogenic itself, can serve as a broad analogue for fluvial and lacustrine sedimentary terrains on Mars, particularly where discrete sedimentary deposits containing alteration phases lie within predominantly basaltic terrain. In addition, these sites also act as specific glaciolacustrine and glaciofluvial systems.

With relevance to the preservation and detection of organic material within fluvial-lacustrine sediments, the glaciovolcanic lacustrine systems outlined here can serve as a model as to how well organic matter can be preserved within environments with low biological productivity, and, at least for the active systems, where primary production is predominantly driven by chemolithoautotrophy rather than phototrophy. In addition, given the necessity to confidently distinguish between authigenic and detrital clay (and other alteration) minerals via *in situ* exploration [20], such sites can be used to test the interpretation of data products acquired from future prototypes and breadboard instruments, where both authigenic (e.g., glaciovolcanic hydrothermal lakes) and detrital alteration minerals (e.g., fluvial sediments) can be investigated within a connected system (*i.e.*, tracing mineral formation and transport from source to sink). A summary of site-specific analogue science is summarized in Table 1. For example, the sedimentary stratigraphic exposures at Gígjökulslón, Brúarjökull proglacial region, and Kverkjökull lend themselves well to field-testing imaging instrumentation in particular, where the scientific objectives are to incorporate geological context and palaeoenvironmental interpretation of lithologies from wide-angle, high resolution, 2D and 3D image data. This is especially true when geomorphological features are important in assessing past habitability, such as grain size, roundness, and sorting (e.g., [72]). Likewise, the assessment of palaeohabitability from rover- or lander-based remote sensing datasets such as those produced by Pancam/Mastcam, MAHLI, and ChemCam instruments [75] can be fine-tuned at such sites where specific stratigraphic horizons may need to be targeted (e.g., when searching for biosignatures) within sedimentary exposures comprising different lithofacies. For example fluvial sediments deposited along the Skaftá river during the periodic catastrophic draining of the Skaftá subglacial lakes would be an ideal target to test for the preservation of organic carbon fixed by the chemolithotrophic communities residing within the subglacial lakes of Vatnajökull, while glaciolacustrine and fluvial stratigraphy within the Brúarjökull proglacial region and the Fljótsdalur and Jökuldalur valleys can be used for testing spectroscopic instrumentation and the detection of discrete mineralogical horizons. To this end, given the range of grainsize, sedimentary fabrics, and mineralogy present within different fluvial and lacustrine lithofacies sourced predominantly from basaltic protoliths, the preservation of organics as a function of depositional time (*i.e.*, rapid, slow), grainsize (*i.e.*, fine or coarse), and mineralogy (*i.e.*, detrital basalt or alteration phases) warrants investigation. The same applies to inorganic biosignatures, such as stable isotope fractionation values of sulfur produced through microbial sulfur reduction and preserved by sedimentary pyrite grains within lacustrine sediments [41].

Finally, the range of coarse to fine-grained lithologies at all these sites make these them suitable for testing sampling and sample manipulation instrumentation (such as corers, scoops, drills, grinders, sieves and crushers), particularly where instruments have to cope with a local mixture of different grainsize and matrix components. In this sense, large-scale sites at Gígjökulslón and the proglacial regions of Brúarjökull and Kverkjökull that incorporate a variety of fine to coarse-grained lithofacies can be used not just for discrete instrument testing, but also for testing mission operations and payload deployment.
